# Environment Monitoring of Rose Crops Greenhouse Based on Autonomous Vehicles with a WSN and Data Analysis

**DOI:** 10.3390/s20205905

**Published:** 2020-10-19

**Authors:** Paul D. Rosero-Montalvo, Vanessa C. Erazo-Chamorro, Vivian F. López-Batista, María N. Moreno-García, Diego H. Peluffo-Ordóñez

**Affiliations:** 1 Department of Computer Science and Automatics, University of Salamanca, 37008 Salamanca, Spain; vivian@usal.es (V.F.L.-B.); mmg@usal.es (M.N.M.-G.); 2 Department of Applied Sciences, Universidad Técnica del Norte, Ibarra 100150, Ecuador; 3 Department of Technologies, Instituto Tecnológico Superior 17 de Julio, Urcuquí 100650, Ecuador; verazo@ist17dejulio.edu.ec; 4 School of Mathematical and Computational Sciences, Yachay Tech University, Urcuquí 100650, Ecuador; 5 Department of Engineering Corporación Universitaria Autónoma de Nariño, Pasto 520002, Colombia; 6 Intelligence for Embedded Systems—Research Line, SDAS Researh Group, Ibarra 100150, Ecuador

**Keywords:** ambient intelligence, autonomous vehicles, monitoring systems, roses crops, wireless sensor networks

## Abstract

This work presents a monitoring system for the environmental conditions of rose flower-cultivation in greenhouses. Its main objective is to improve the quality of the crops while regulating the production time. To this end, a system consisting of autonomous quadruped vehicles connected with a wireless sensor network (WSN) is developed, which supports the decision-making on type of action to be carried out in a greenhouse to maintain the appropriate environmental conditions for rose cultivation. A data analysis process was carried out, aimed at designing an in-situ intelligent system able to make proper decisions regarding the cultivation process. This process involves stages for balancing data, prototype selection, and supervised classification. The proposed system produces a significant reduction of data in the training set obtained by the WSN while reaching a high classification performance in real conditions—amounting to 90% and 97.5%, respectively. As a remarkable outcome, it is also provided an approach to ensure correct planning and selection of routes for the autonomous vehicle through the global positioning system.

## 1. Introduction

Rose cultivation has a great impact on the economy of Ecuador, as theses flowers are exported and cover 9% of the world’s market. Rose cultivation brings approximately 500 million dollars to the national budget and covers 8000 hectares in the country [1]. With the growing demand for flower farming production, a natural environment is not always the optimum to achieve the necessary crop requirements. Extreme conditions such as direct sun exposure, hail, diseases, and pests can seriously affect the quality of the product and the volume of production [2]. For this reason, large-scale greenhouses are becoming increasingly more popular, because they can modify the environmental conditions of the interior by means of lights, ventilation, heating, among others. Thus, crop production cycles can be planned based on market needs [3,4].

Additionally, floriculture production must be carried out in an efficient and sustainable manner, causing the least negative impact on the environment. Bearing this in mind, the use of technology allows for innovating processes and decisions based on previously collected information. In this manner, the use of agricultural resources and supplies can be improved. However, the process of data acquisition requires great effort, especially when it comes to the implementation of connections and the distribution of sensors  [5]. In some cases, these systems are made using cables and can be complex and expensive. Furthermore, it should be possible to modify the location of the points of measurement according to the particular needs of the crop. Due to their easy implementation and increased mobility, wireless sensor networks (WSN) are an alternative in this respect. A WSN is made up of nodes that have the ability to acquire data and send such data by means of wireless protocols [6,7]. Likewise, WSNs are low-cost, low current-consumption systems, and allow for different types of networks and more flexibility in the exchange of information. As a result, they are efficient electronic systems that can cover large growing spaces. Within the greenhouses, it is necessary to use several WSN nodes that help get reliable data to represent the state of the plant and the preventive actions that can be taken to improve production [5,8]. In addition, the implementation of autonomous-vehicles allows for the mobility of the WSN node, as they can select routes and avoid obstacles [9]. Next, the WSN node will be able to collect data from the entire crop and by means of a GPS module, store said information at its respective location. As a result, a robust data analysis methodology can be implemented that can be compiled in each WSN node in order to make decisions, learn from external stimuli, and adapt to changes [10,11].

The parameters needed for the proper development of rose plants are relative humidity, temperature, electrical conductivity, precipitation, CO${}_{2}$, and light intensity [12]. In many cases, these are not taken into account in the implementation of irrigation systems. In fact, the systems are generally based on timers or criteria based on the experiences of the people in charge of the production of roses [13]. Due to this, the ground within a greenhouse does not have homogeneous conditions[14]. Consequently, rose plants may modify their functional cycle, and obtain buds in very early stages, which are vulnerable to the use of fungicides with humid and foliar acids. Therefore, in some cases, the harvest time of the plant is modified and causes delays during the seasons of greater commercialization [15].

The proposed system is made up of a WSN implemented on quadruped autonomous vehicle moving inside a greenhouse. Firstly, the WSN has a set of sensors that monitors relative humidity, CO${}_{2}$, room temperature, light quantity, and soil moisture. This is done in such a manner that the entire data analysis process can be incorporated and executed within each WSN node with a low consumption of computational resources. Secondly, the quadruped vehicle is designed to avoid collisions and be able to move in the greenhouse through the global positioning system (GPS). For this, there are established points within the greenhouse considered as arrival objectives. As a result of this system, there is a 90% reduction in the training data matrix acquired during the entire rose growing cycle for the classification algorithm training, which obtained a performance of 98% under simulated conditions and 97.5% in actual operation.

The rest of the document is structured as follows: Section 2 shows related works. Section 3 presents the system’s design. Section 4 shows the data analysis proposal for the implementation of machine learning algorithms. The tests and results are shown in Section 5. Finally, Section 6 highlights the most relevant conclusions and future works.

## 2. Related Works

Typically, roses meant for export are cultivated by means of precision agriculture, which includes the following stages: (i) data collection, (ii) processing, (iii) data analysis, and (iv) decision making. In chronological form, they are presented to the most relevant in the different years. Salleh et al.  [2] presents a WSN for monitoring environmental conditions in greenhouses (2013). The nodes of the WSN have solar cells and Zigbee modules for communication. Their results focus on optimizing system resources to extend the system battery. On the other hand, in the same year, Pekosawski et al. [5] present a similar application of WSN but with the objective of analyzing gases such as CO, CO${}_{2}$, and CH${}_{4}$. Similarly, their application uses Zigbee modules. In the following year, a trend to perform irrigation actuators takes place as explained in Mat et al. [16]. This work describes software and hardware components for the activation of water and fertilizers in a controlled way. Then, in 2015, Liang-Ying et al. [7] used GPRS systems to send data remotely and thus avoid having a local network by sending the information to a digital repository. In 2016, Liu et al. [17] introduced the first approach to data analysis through the use of prediction models. Furthermore, in 2017, Sampaio et al. [18] presented an alternative consisting in counting the hierarchical sensor nodes for data submission priority.

In 2018, Puspitasari et al. [19] presented the first real-time applications using WSNs with a minimum bit-sending error rate. The same year, Shinde et al. [15] presented an approach focused on the structure of a WSN for the Internet of Things (IoT), by sending packets in new lightweight protocols under the TCP/IP model. Finally, in 2019, Durmus et al. [20] presented a WSN with integrated mobile robots and data collection through IoT.

Given the works cited above, it can be stated that the WSN tends to use IoT connection protocols and evaluates the mobility of systems for the homogeneous acquisition of data in the greenhouse, where the optimization of resources is becoming a much debated problem. However, most cases focus on laboratory scale solutions, which—given their dimensions—are able to be processed by a traditional WSN. Furthermore, the electronic system has no incorporated filtering and data coupling steps to minimize noise from the non-linearity of the electronic elements that make-up the sensors and carry out the conversion of a physical parameter to an electrical signal. In this respect, most of the investigations reviewed have no clean or prepared dataset that can be directly and successfully processed through decision-making systems (e.g., machine learning algorithms). In addition, they do not address the effect that the implementation of this technology has on their products. This is in fact the rationale behind the proposed system, as the developed embedded systems are intended to keep a good trade-off between the machine learning algorithms requirements and computational resources. Consequently, the proposed data analysis methodology is of great importance in demonstrating the advantages of implementing this system in real conditions.

## 3. Electronic Design

**Figure 1:**
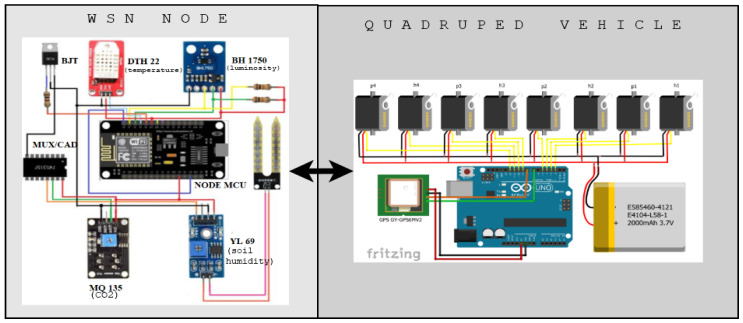
Schematic diagram of the proposed embedded systems for both the wireless sensor network (WSN) node (left side) and the quadruped vehicle (right).

This section outlines the electronic design of the vehicle fitted with sensors as well as the sensor network, which is—as a whole—referred to as the system. By design, the development of the proposed system consists of three main stages: sensor network design (Section 3.1), design of the quadruped autonomous vehicle (Section 3.2), and its motion-planning alongside the WSN topology design (Section 3.3). A schematic diagram of the proposed system is shown in Figure 1.

### 3.1. Sensor Network Design

The environmental parameters within a greenhouse for a rose plant are temperature, luminosity, ground humidity, relative humidity, and CO${}_{2}$. Each of them influences the proper growth of the rose [21]. Consequently, if the temperature is lowered below the recommended value, this can cause irregularities in the rose blossom; if it is higher, the flowers increase in number, but their quality is affected. In addition, light intensity, relative humidity, and CO${}_{2}$ directly influence the photosynthetic process. Likewise, ground humidity defines the amount of water contained in unit volume of soil. If its value is not adequate, there is less chance of increasing evapotranspiration (loss of water due to heat and crop perspiration) [22,23]. The optimal values for environment variables are: room temperature 17–28 °C, light intensity 440 lx–680 lx, ground humidity 55–65%, relative humidity 70–80%, and CO${}_{2}$ 800 ppm–900 ppm [23].

For the adequate development of the WSN, the selection of the sensors must be done based on strictly-defined operating requirements. Among the main ones that were taken into account are reliability, precision, availability, ease of use, and scalability. In addition, for the selection of the WSN processor system, wireless connectivity and the protocol used were considered [14]. As a result, the sensors chosen were: DHT 22 (relative humidity and temperature), MQ 135 (CO${}_{2}$), YL 69 (soil humidity), BH 1750 (luminosity). Finally, the NodeMCu was selected as a processor for its communication to WiFi networks. This way, data can be flexibly sent within an AD-HOC network created by the same devices, a greenhouse network, or stored in the cloud. As additional elements, an analog–digital multiplexer/converter (8 channels multiplexed at 1 output) is required in order to be able to read several sensors. This is because the NodeMCu only has a digital–analog converter. Additionally, a bipolar junction transistor (BJT-NPN) that works in cut and saturation is used to activate the sensors. For the power supply of the system, there is an LiPo type battery with a self-charging system that works by means of a battery manager and solar panels. Based on the datasheets of each of the electronic elements used, there is an approximate total consumption of 260 milliamps. The battery manager used is Lio Rider which supplies power from a solar panel until it reaches 400 milliamps. If this value is not enough, the battery can also get charged via USB-port.

### 3.2. Quadruped Vehicle Design

There is a wide variety of methodologies for the uniform sampling of crops. In relation to the proposed system, a grid-shaped path is defined, since it is effective with rose crops planted in a greenhouse and the vehicle can function properly  [24].

**Figure 2:**
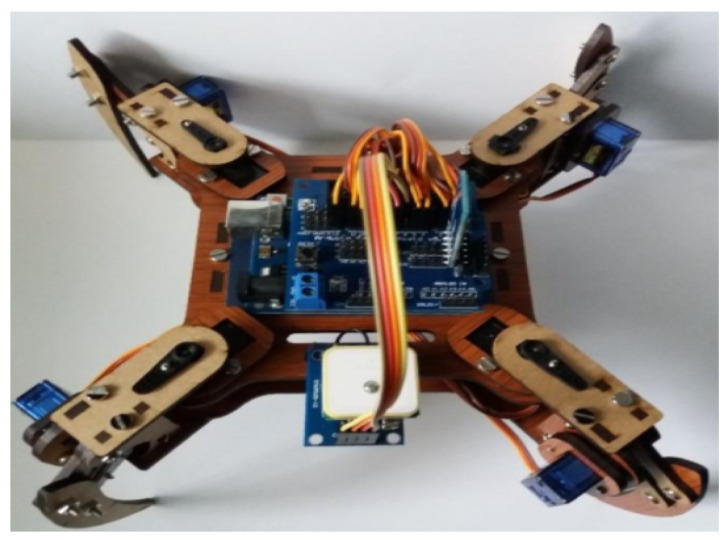
Quadruped vehicle in charge of collecting the greenhouse information.

The quadruped vehicle uses open hardware of the mePed version [25]. Its files of dimensions and cut-off points are free to modify in size and usability. In this case, the scale was increased 2.25 to have servo motors of greater force (8 in total). In addition, for route planning, it has a Neo 6m (GPS) module for its location, an MPU-6050 sensor (accelerometer) to determine its orientation, a 3000-milliamperes LiPo battery, and an ArduinoUno as a processor. The armed vehicle used for testing is shown in Figure 2.

For notation purposes, henceforth the term “marks” is used to refer to the vehicle’s turn, while “sub-marks” accounts for sampling. Inside of the greenhouse, the vehicle operation can be divided into two stages. The first stage is the movement and location of marks and sub-marks. Each of them is marked with their coordinates where the vehicle must arrive.

**Figure 3:**
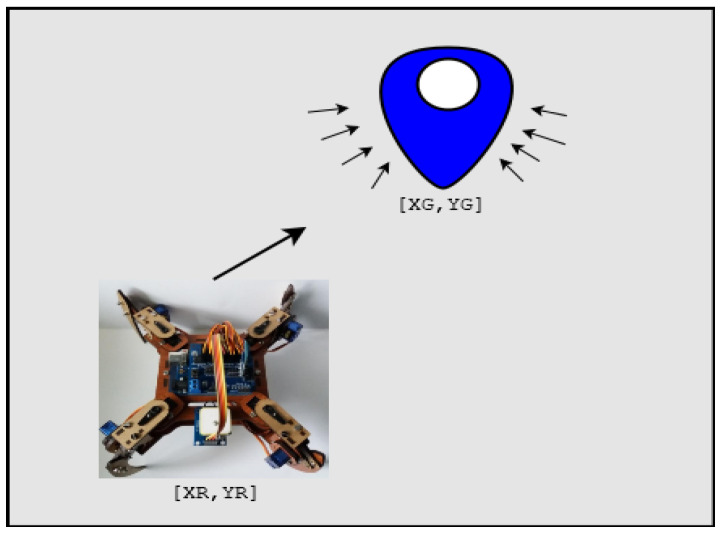
Potential field action vectors, white circle (goal), and blue region (potential field).

To achieve this, we use the active potential field, which means that an action vector is defined to direct towards the desired mark. This criterion has two imaginary fields (attractive potential and repulsive potential). The result is a simple real-time route planning approach. The action vector is found by applying a scalar based on the potential field to the position of the vehicle and then the gradient of that function [9]. Figure 3 shows attractive potential field action vectors pointing to the goal and theirs Equations (1) and (2).

For the following statements, the following notation is considered: $\left[X_{G},Y_{G}\right]$ as the position of the goal, *r* as the radius of the goal, $\left[X_{R},Y_{R}\right]$ as the position of the vehicle, *s* as the size of the goal’s area of influence, and α as the strength of the attractive field (α>0). In this context, $\left[\nabla x,\nabla y\right]$ are the coordinates of the autonomous vehicle’s movements.

The distance *d* from the mark to the autonomous vehicle is:

(1) $$\begin{array}{c} d={\left[{\left(X_{G}-X_{R}\right)}^{2}+{\left(Y_{G}-Y_{R}\right)}^{2}\right]}^{\frac{1}{2}}, \end{array}$$

while the angle θ in between the two, is given by:

(2) $$\begin{array}{c} \theta =tan^{1}\left(\frac{Y_{G}-Y_{R}}{X_{G}-X_{R}}\right). \end{array}$$

As a result, the following planning rules are feasible:

- If d<r, then ∇x=∇y=0.
- If r≤d≤s+r, then ∇x=α(d−r)cos(θ) and ∇y=α(d−r)sin(θ).
- If d>s+r, then ∇x=αscos(θ) and ∇y=αssin(θ)

**Figure 4:**
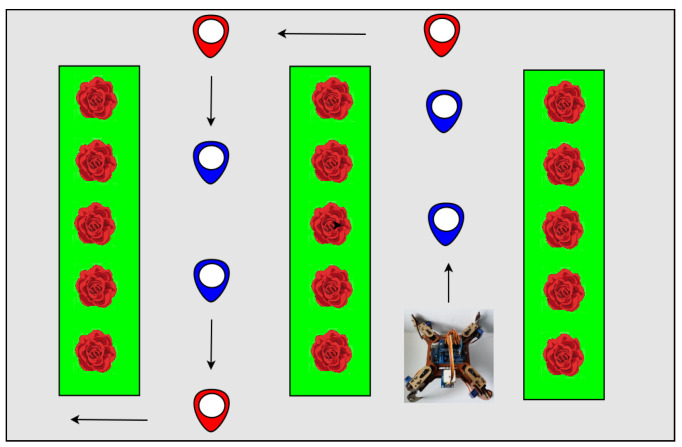
Vehicle movements inside to greenhouses, marks: red, sub-marks: blue.

The second stage is soil moisture acquisition, which is carried out by the proper sensor. To do this, the vehicle reaches every sub marks, the sensor located below the chassis can be inserted with the weight of the vehicle itself. Subsequently, the system sends an activation flag to the WSN to acquire data for the rest for the sensors. This process occurs every 3 m. Figure 4 shows the route of the vehicle with the established marks and sub marks. It should be noted that the cultivation area is 50 m long and 30 m wide and there are 1-meter wide cultivation beds located 60 cm apart.

### 3.3. WSN Topology

**Figure 5:**
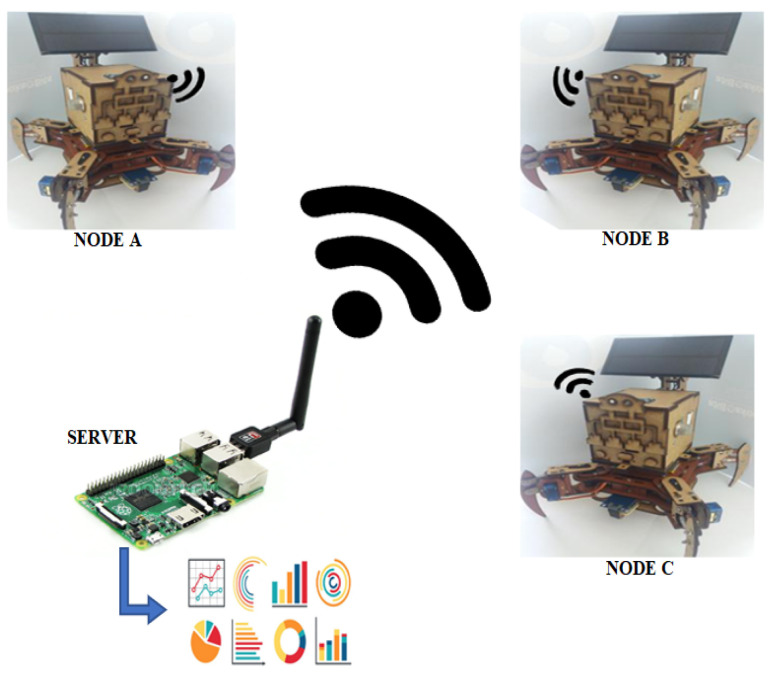
WSN topology with WiFi transfer data protocol and Raspberry Pi as a server.

The proposed WSN has three autonomous nodes that use the sub-marks to cover the entire cultivated surface. This assignment of marks are done through the main node, which is in turn in charge of generating the routes and dividing the system based on all the previously acquired coordinates of the greenhouse dimensions. To do this, a point to multi-point topology is established, where the central node is a Raspberry Pi 4. A desktop computer or server is not used as a central node because the system requires a certain mobility. The process of sending data is carried out through WiFi communication, since all nodes handle this protocol. The proposed WSN topology is shown in Figure 5.

## 4. Data Analysis

In this work, the data analysis is focused on reducing the instances of a high volume data matrix to achieve an improved matrix in order to be used for the training of a supervised classification algorithm and that this can be implemented in each WSN node (as seen in Section 4.1). In such vein, whether reaching a classification performance similar to that obtained when using the high-volume matrix: the more reduced the data matrix, the better the data representation technique. In this way, each node can make its own decision and send this information to the central node. Therefore, the aim was to find the least amount of data representing the studied phenomenon. There exists a wide range of different criteria to perform the task of selecting characteristics. In this work, data balancing (Section 4.2) is implemented to avoid over-training regarding majority-class samples. In addition, a prototype selection technique (Section 4.3) is used in order to eliminate data that does not provide important information to the classifier [26]. Finally, a comparison of the classical supervised classification criteria (Section 4.4) is made to choose the appropriate algorithm that maintains a high compromise between the classification performance and the computational cost that the decision represents.

### 4.1. Data Acquisition

An acquisition stage consisting of coupling and filtering data is proposed with the aim of eliminating reading errors. Following from this, the DC-voltage components that can occur due to the non-linearity of the sensors are eliminated. In this sense, the average filter is preferred, which works as follows: It samples by windows of size *d* on a input vector $x=[x_{j}]$ to find the average per window to yield a single point of smoothing vector $y=[y_{k}]$. This filtering process is known as windowing or dynamic average and can be expressed as:

(3) $$\begin{array}{c} y_{k}={\left(2n+1\right)}^{-1}\sum_{i=k-d}^{k+d}x_{j}, \end{array}$$

where $x=(x_{1},\ldots ,x{}_{L_{x}})$ is the input signal (50 samples are acquired at each established monitoring mark), $y=(y_{1},\ldots ,y_{L_{y}})$ is the filtered signal, *d* is the window size, and $L_{x}$ and $L_{y}$ are respectively the input and filtered signal lengths. To account for a reduction in the computational resources usage, we experimentally define d=10.

**Figure 6:**
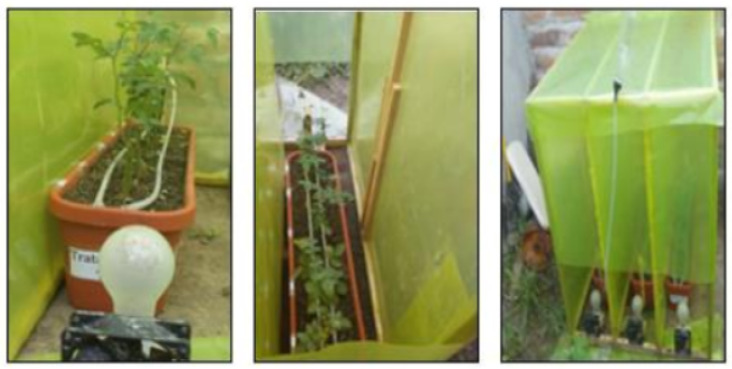
Rose crop micro-environments.

In order to establish the different environmental conditions of the crop, we created micro-environments that represent the both appropriate and harmful conditions within a greenhouse. The construction of the three micro-environments is shown in Figure 6.

By using such a technique, the environment can be changed quickly without affecting a large harvest. It was possible to increase the amount of irrigation water, vary the ambient temperature, and have different amounts of CO${}_{2}$ to observe their effect on roses throughout the entire growth process until the harvesting stage. At the end of this process, we obtained the classes for the database, and the subsequent learning of the system. Table 1 relates the labels of each class, their respective representation, and the modification made in their treatment to achieve different cultivation conditions. It should be considered that a greenhouse where roses are grown should have a drip irrigation system, ventilation curtains, and sunshine [23].

After a cycle of rose crops (approximately 5 months), the matrix of data obtained is ***Y*** $\in R^{m\times n}$, where *m* is the number of examples and *n* is the number of measured environmental variables (sensors). Meanwhile, $L=\left[\ell_{i}\right]\in R^{m\times 1}$ is the label vector, with $\ell_{i}\in \left\{1,\ldots ,4\right\}$, i∈{1,…,m}, m=2500, and n=5. It is important to highlight the fact that the original input data matrix cannot be stored into the WSN node because of its large size. Given the aim of improving representation and maintaining relevance, it is required that a data representation stage be carried out in advance.

### 4.2. Data Balancing

Under a criterion of supervised classification, an algorithm learns in relation to past instances. As a result of this, if there is a class with a greater number of data than the rest in a high volume of information, the outcome may result in an over-trained classifier which may favor the prediction of a specific label (class) in new incoming data. As a result, the classifier loses its sensitivity in complex data. The data matrix $Y\in R^{m\times n}$ has this drawback. For the correct label assignment, the micro-environments generated for the study of rose cultivation, some of them (labels 1 and 4) greatly affected the growth of the plant. For this reason, the sampling time is shorter than that of the rest of the micro-environments (about half of the rest of the labels). In addition, despite the fact that similar sampling times were established, due to the effects of calibration times and the type of data to be processed within the signal smoothing algorithms of each sensor, their response time is varied. Therefore, according to [27], it indicates that the Kennard–Stone algorithm represents the best option in data obtained by sensors. This means that each label has the same samples in the training set.

### 4.3. Prototype Selection

A WSN has limited computing resources; the greater the amount of data to be processed, the longer its response time and the more affected the battery life. On the other hand, if all the data are used, there is a high possibility that many of them do not provide meaningful information to the classifier, which will reduce its decision capacity due to the model overfitting. In this sense, the prototype selection criterion (PS) is based on the concept that proper data pre-processing can reduce the size of the training matrix while maintaining the predictive ability of the algorithm. This does not affect the intrinsic knowledge initially stored.

PS algorithms are related to 3 approaches. We choose the most representative of each of them, namely: Condensation: Condensed Nearest Neighbor (CNN), Reduced Nearest Neighbor (RNN), and Selective Nearest Neighbor (SNN). Edition: Edited Nearest Neighbor (ENN), All-k Edited Nearest Neighbors (AENN), Iterative Partitioning Filter Method (IPF), and Hybrid: Decremental Reduction Optimization Procedures 2 (DROP 2), Decremental Reduction Optimization Procedures 3 (DROP3), and Iterative Noise Filter Method based on the Fusion of Classes (INFFC) [28].

### 4.4. Classification Algorithms

Classification algorithms can learn from different training criteria, such as: (i) distance-based (k-NN), (ii) density-based, (iii) model-based, and (iv) heuristic criteria. In this sense, we have used and evaluated a representative algorithm for each criterion, as follows: (i) k-NN, (ii) Bayesian classifier, (iii) support-vector-machines-based classifier (SVM), and (iv) decision tree  [29].

## 5. Experimental Setup and Results

This section gathers the obtained experimental results, which are structured as follows: The embedded system developed in this work is presented in Section 4.4. To assess the behavior of each stage, the reduction of training data are discussed in Section 5.2. Then, the classification results are described in Section 5.3. Finally, the results of the implementation of the data analysis within the autonomous vehicle with the WSN and its operation of real conditions are shown in Section 5.4.

### 5.1. Developed WSN

**Figure 7:**
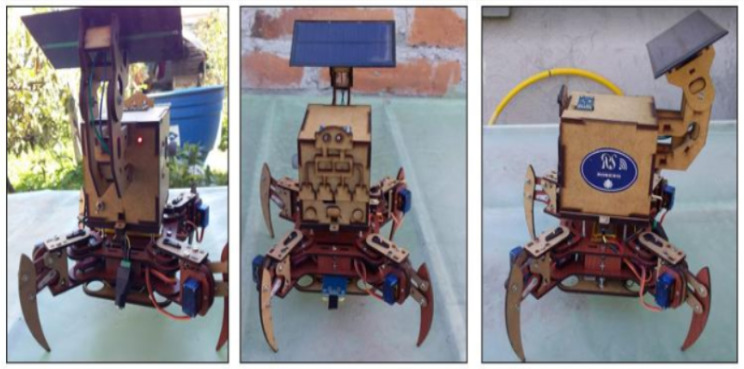
Implementation of the nodes onto the autonomous vehicle.

With the WSN nodes setting (three in total), they were placed inside a housing made of chipboard material, this is due to its easy cutting, design and durability. In addition, function LEDs were implemented for each WSN node. When the system is running and data are detected, the green led shines; when the battery is running low, the red led is turned on. The solar panel has been placed at the top so that it can receive sunlight in the greenhouse. When there is no sunlight, it can switch to the normal battery. In Figure 7, the complete system is shown from the different viewpoints.

Once the WSN nodes are configured and running, the connection to the autonomous vehicle shall be implemented. These communicate through serial communication. In this way, when the autonomous vehicle reaches its mark, it sends an activation bit to the WSN to acquire the data coming from the sensors. The vehicle tilts down to bury the humidity sensor and sends that data with its GPS location to the WSN. Then, the WSN sends an end of process bit. Subsequently, the vehicle moves again until it finds another GPS mark.

With the autonomous vehicle assembled and all its parts configured, the performance tests were carried out in order to establish two aspects. The first is the battery life, where the vehicle worked without energy-saving modes in the WSN and with all the quadruped vehicle sensors running permanently. As a result, the autonomous vehicle battery lasted approximately 8 h and the WSN ones for 6 h. The second aspect refers to the sleep mode schedules implemented in the WSN. As a result of these modules, the WSN worked normally for 14 h, without the solar panel function. When it supplied extra current with the solar panel when the battery is wasted, the WSN works weeks without been turned off.

### 5.2. Training Matrix Reduction

By implementing the Kennard–Stone algorithm, the matrix $Y\in R^{m\times n}$ was reduced to $X\in R^{p\times n}$, where p=1600 (400 data per label). Subsequently, the matrix $X\in R^{p\times n}$ was divided into training set (80%) and test set (20%). With the new base generated, a comparison of the PS algorithms is made with the processing time metrics, removed instances and their redundant data elimination average in training set. Table 2 shows the data obtained. The CNN algorithm proved to have the best behavior in relation to the processing time and the elimination of instances. As a result, a new matrix $Z\in R^{s\times n}$ is defined, where s=128. This process is executed by the Raspberry pi 4.

### 5.3. Classification Performance

A comparison of the supervised classification algorithms is carried out to determine the most appropriate ones. This is done with the classification models trained with the least-sized dataset provided by the before-performed prototype selection stage. At first, the performance of each of them is established with the training set $X\in R^{p\times n}$ (data balanced for each label). Subsequently, the matrix $Z\in R^{s\times n}$ (Prototype selection training set) is used to observe which algorithm best represents the intrinsic knowledge of the data. It should be taken into consideration that this performance is carried out with the test of X (20%), which corresponds to 320 instances. In addition, SVM has some kernels that can be used; in this case, they were implemented: polynomial, sigmoid, and radial. Table 3 shows the performance of each algorithm with respect to the two data matrix.

As can be appreciated, the k-NN algorithm reaches a higher classification performance. Therefore, different metrics to evaluate the classification performance are computed, namely: average (acc), error(err), sensitivity (sn), specificity (sp), precision (p), and area below the operational characteristic curve of the AUC receiver. These are good discriminators of selection of classification algorithms [29]. In Table 4, the algorithm k-NN is observed with the different metrics in relation to each classification label.

**Figure 8:**
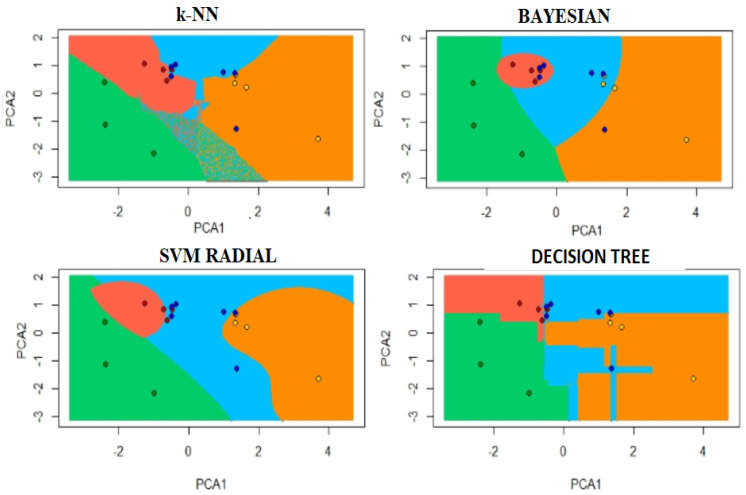
Decision boundaries per label of each classifier. Class 1: green color, class 2: red color, class 3: blue color and class 4: orange color. $X_{axis}$= Principal component 1. $Y_{axis}$= Principal component 2.

In order to obtain a graphical representation of each classification algorithm, the prediction of new instances was reduced to two dimensions through the Principal Component Analysis. This algorithm is used since it readily enables the data distribution representation in a lower dimension. Consequently, the decision edges of each label are colored. The effect of separation caused by every considered classifier can be observed in Figure 8 displayed over a lower-dimensional space for visualization purposes.

For the purposes of this analysis, we define a CNN as a redundant data elimination algorithm and a k-NN as a classification algorithm.

### 5.4. Autonomous Vehicle with WSN Implemented

**Figure 9:**
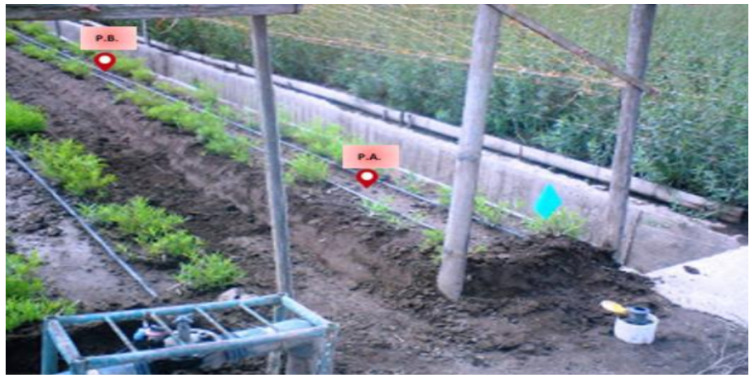
Sub-mark acquisition inside the greenhouse area.

First, we observe how marks and sub-marks are approached within the crop. With this, you can plan the routes of the vehicles and set their respective turn sequences. Figure 9 shows the rose crops at the beginning of their cycle and the acquisition of marks in a greenhouse.

The WSN node has the data stored in a matrix ***Z***; when it receives new data from the sensors, k-NN goes into operation and decides the class (set of action). Then, it receives the location of the quadruped and sends all the information in a characters vector by means a WiFi network created in the place. Subsequently, this data passes through the CNN algorithm, which verifies the valid information that can improve the training matrix. If the algorithm decides so, it is stored in the matrix ***Z***; otherwise, only the sending process is performed.

**Figure 10:**
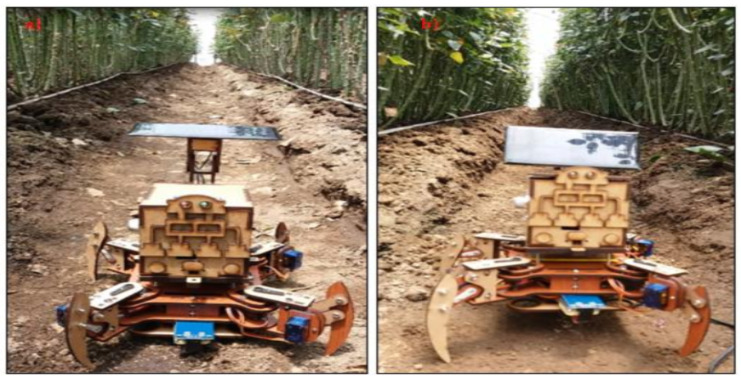
Autonomous system with WSN.

Consequently, we set specific times for the compliance of routes and battery saving modes in the sensors used. As a result, the quadruped performs 4 routes a day (morning, afternoon, night, and early morning), and each route takes approximately 1 h. In addition, given its energy-saving modes, the WSN was activated only when the vehicle sent a warning about a mark that it found. For this reason, the system as a whole can operate continuously for a duration of 3 days for the quadruped and 9 days for the WSN. By having the 3 nodes working simultaneously, these vehicles can cover between 80 to 100 square meters of crops daily. The operation of the autonomous vehicle in the greenhouse is shown in Figure 10.

As a second point, it is the correct decision of the system and its assessment with respect to the chosen action by experts (people holding practical expertise and background on crops and environmental measurement equipment). These tests were performed to define the correct action of the system in the different locations of the greenhouse. Forty functioning tests were performed to assess the decision-making capacity of the system. The system had 97.5% success in the actions taken inside the greenhouse. As a result, roses have higher stem growth and better leafiness in cultivation. In terms of return on investment, the implementation of this system had an initial margin of increasing 5% the net profit from the crop. This is related to the lower consumption of water (15% percent), less use of pesticides (8% percent), and the result of the sale of roses (3%). It should be noted that from an economic point of view, the implementation of autonomous vehicles is well below the cost of other monitoring systems within the Ecuador–Colombia market. In addition, it was possible to verify that the greenhouses do not have homogeneous environmental conditions, there are certain sectors that due to their location in relation to the sun or distribution of the irrigation system have certain moisture deficiencies that cause less amount of ${\mathrm{CO}}_{2}$ for the photosynthetic process of the plant.

Regarding the movement of the quadruped, the error in reaching each established mark has a variability of 0.35 mrts. This is due to slight variations in the collecting of GPS data related to the turn towards the other marks in the rose growing-beds. The turning angle had an error of 2 degrees in total. However, this problem is corrected by searching for the next mark inside the greenhouse.

## 6. Conclusions and Future Work

Related to the selection of the sensors in conjunction with the autonomous vehicle, they provided adequate operation by meeting the established marks and sub-marks inside the greenhouse. With this, the data acquisition process provided information about the crop for the implementation of the data analysis stage. This is thanks to filtering reading errors by means of data smoothing.

The WSN with autonomous quadruped vehicles fulfills the objective of providing information on the cultivation of roses by sectors within the greenhouse. Consequently, the planned scheme for data analysis was adequate, since it allowed a significant reduction of redundant data and computationally lightweight classification algorithms that can be implemented in WSN nodes with limited resources. In addition, it allows for the collection of a large amount of information that can be useful for years to come, helping farmers modify their techniques with respect to climate change.

With the autonomous vehicle, it was possible to properly arrange the growing cycles of roses. In this way, we propose a new approach to the design and construction of greenhouses, which allows for the flexibility that the crop needs (fans and floodgates in different locations, not centralized). This way, a more extensive analysis can be made with regard to change of environmental parameters in order to find optimal growth values and improve product quality.

As far as future work is concerned, we recommend exploring the use of batteries, their charge, and weight, thus ensuring better design and movement. In addition, it should be noted that the quadruped could be mobilized on irregular ground, but it had balance issues and struggled depending on the distance from the planned route. With this in mind, we suggest using other mechanisms as tank chassis.

## Figures and Tables

**Table 1:** Roses crop micro-environment labeling.

Label	Action	Treatment
1	Watering	Water each 8 days and curtains open only in the morning.
2	No action	Water each 4 days, curtains open in the morning and closed at night.
3	Open curtains	Water each 2 days with closed curtains.
4	Close curtains	Water each 4 days, curtains open.

**Table 2:** Prototype selection algorithms analysis.

PS Algorithm	Proc. Time (s)	Remv. Inst	% of Remv. Inst
AENN	24.02	16	1.25
BBN R	30.15	22	1.72
CNN	23.76	1152	90.0
DROP1	728.67	1120	87.5
DROP3	830.18	1120	87.5
ENN	6.14	32	2.5
RNN	27.23	1152	90.0

**Table 3:** Performance of the considered classification algorithms.

Classification Algorithms	Performance (%) on Matrix $X_{\left(1280\times 5\right)}$	Performance (%) on Matrix $Z_{\left(128\times 5\right)}$
k-NN	98.33	98.33
Class. Bayesian	95.27	95.27
Decision tree	95.27	93.44
SVM polynomial	95.27	95.08
SVM radial	95.08	95.08
SVM sigmoid	93.44	88.52

**Table 4:** Performance values with k-NN.

Label	Acc (%)	Err (%)	Sn (%)	Sp (%)	P (%)	AUC (%)
1	100	0	100	100	100	100
2	96	3	87	97	85	92
3	90	10	57	94	57	90
4	93	7	84	95	84	90
**Average**	94	5	82	97	82	89
